# When Accelerated MD Program Is Not the Right Path: The Why and How to Support Accelerated Learners in the Transition to the 4-Year Program

**DOI:** 10.1007/s40670-025-02328-5

**Published:** 2025-02-22

**Authors:** Allison Macerollo, Sally A. Santen, Judith Brenner, Joan Cangiarella, Alicia Gonzalez-Flores, Betsy Goebel Jones, Shou Ling Leong, Caroline Roberts, Christin Traba, Christina M. Vitto, Lisa Strano-Paul

**Affiliations:** 1 Department of Family and Community Medicine, The Ohio State University College of Medicine, Columbus, OH USA; 2 Virginia Commonwealth School of Medicine, Richmond, VA USA; 3 Present Address: University of Cincinnati College of Medicine, Cincinnati, OH USA; 4 New York University Grossman Long Island School of Medicine, Mineola, NY USA; 5 NYU Grossman School of Medicine, New York, NY USA; 6 University of California Davis School of Medicine, Davis, CA USA; 7 Texas Tech University Health Sciences Center School of Medicine, Lubbock, TX USA; 8 Pennsylvania State University College of Medicine, Hershey, PA USA; 9 Department of Family Medicine, University of North Carolina - Chapel Hill School of Medicine, Chapel Hill, NC USA; 10 Rutgers New Jersey Medical School, Newark, NJ USA; 11 Virginia Commonwealth University, Richmond, VA USA; 12 Renaissance School of Medicine at Stony, Brook University, Stony Brook, NY USA

**Keywords:** Accelerated curriculum, Program evaluation, Student Support

## Abstract

Accelerated 3-year medical school programs (A3YP) allow students to complete medical school in 3 years rather than the traditional 4-year program (4YP). This paper describes the perspective of 14 A3YPs, exploring the rate of and reasons for transition out of an accelerated pathway into a traditional 4YP. As of 2023, 19% of students in A3YP transitioned to 4YP. The authors provide practical recommendations to guide transitions based on pooled experiences and expert consensus from members of the Consortium of Accelerated Medical Pathway Programs (CAMPP). Having clear policies that define academic, clinical, and professional expectations and processes to guide transition is important.

Accelerated 3-year medical school programs (A3YPs) allow students to complete medical school in 3 years rather than the traditional 4-year program (4YP) [1–5]. These programs are based on a variety of missions including increasing primary care providers and decreasing debt. The creation of a more efficient medical education program is supported by calls for time variable competency-based education and questionable utility of the fourth year of medical school [6, 7]. A3YPs often come with a directed pathway to a specific residency program. Students are admitted to the A3YP programs prior to matriculation or during the pre-clerkship phase.

Since 2015, the number of schools offering A3YPs have increased substantially, growing from seven programs in 2015 to 32 US programs in 2023. Programs provide benefits to students including debt reduction, directed pathway to residency, enhanced opportunity for early mentorship, and the ability to enter the workforce sooner [8]. Several studies have demonstrated that there are no clear adverse effects related to quality of education, readiness for residency, internship performance, or burnout [9–11].

For many schools, A3YPs have their own selection process and admission to an accelerated pathway occurs prior to matriculation or during the pre-clerkship phase. Selection is usually based on interest in, and evidence of commitment to a chosen specialty and schools require that students in an A3YP meet or exceed the typical admission criteria. Some schools offer a directed pathway into a single specialty, such as family medicine or internal medicine, while others offer acceleration into a variety of specialties. Most commonly, the directed pathway is linked to a graduate medical education (GME) program associated with the student’s medical school. However, because most A3YPs are bound by the National Residency Matching Program (NRMP) agreement, students can also apply to residency programs outside of the home institution. Many A3YPs include GME faculty in the selection of students into the accelerated pathway, and the GME faculty continue as advisors throughout medical school ensuring a smooth undergraduate medical education (UME) to GME transition [4].

Most programs use a combination of longitudinal experiences, such as an elective that may run parallel to the current curriculum and/or summer courses outside of the typical 4-year medical education program to meet the Liaison Committee for Medical Education (LCME) requirement of 130 weeks in a medical educational program. A3YPs focus on competency-based education strategies to ensure that all students meet advancement and graduation requirements for their students. Typical fourth year experiences such as electives, research, and away rotations are eliminated and key rotations, such as acting internships, are moved into the third year.

Student advising and mentorship are integral to all A3YP programs [12–15], as it has been shown that long-term mentorship enhances career selection and satisfaction. Directors of A3YPs serve as advisors to their students and follow their progress closely. In addition, many students have additional career advisors in their chosen specialty.

## Student Transition from Accelerated 3-Year Program to a Traditional 4-Year Program

The A3YP carefully selects students and mentors them through medical school. Nonetheless, circumstances—both academic and personal—may confront students in any pathway or program during their UME years and impact the actual length of their time in medical school. For students in accelerated programs, facing academic and personal circumstances may mean that continuing in the accelerated pathway is not in the best interest of all parties and may in fact hamper a student’s career or personal growth. As additional schools consider creating an A3YP, this perspective provides the opportunity to explore the frequency and reasons for transition out of an accelerated pathway and into the traditional 4YP. Guidance, policies, and procedures to support students if they need to transition out of A3YP are also discussed. This article is based on scholarly work, experiences, and survey information from members of the Consortium of Medical Accelerated Pathway Programs (CAMPP). CAMPP is an organization of medical schools in the USA that have developed 3-year or other accelerated curricula that lead to the MD degree. Representatives from member schools work together to promote, enhance, and evaluate the processes and outcomes related to accelerated medical training. Through collaboration and study, CAMPP seeks to improve physician education by addressing several aspects of accelerated MD degree programs, including financial, regulatory, and competency matters. The group also offers guidance to other medical schools seeking to develop such programs.

Program evaluation of CAMPP participating schools occurred in summer of 2023, with a survey and subsequent follow-up questions of A3YP directors. We included data only from the MD programs with A3YPs that run parallel to a traditional 4YP within the school and that had graduates as of 2023. This resulted in data collection from 14 of the 16 A3YP schools that met criteria of parallel tracks (87.5%) (Table 1, the list of schools in Appendix Table 3). The general survey included questions that were collecting data about the program (data related) and free response. The majority of the questions were data related and focused on beginning date of each program, number of graduates, and how each program is organized. Programs were identified by name, but no specific students were identified. This paper details survey responses centered around on-time graduation versus transitions out of the accelerated pathway and is not inclusive of all data obtained in the original survey. The two main questions we are addressing are detailed in the following paragraphs.

**Table 1:** Numbers of students graduating from 3-year programs and transitioning to 4-year programs and the reasons for transitioning

	Number (%)
Matriculated A3YP	591
Graduated in 3 years	477 (81%)
Transitioned to 4YP	114 (19%)
Reasons for transition	
• Personal	27 (24%)
• Specialty	34 (30%)
• Academic	45 (39%)
• Professionalism	7 (6%)
• Other	1 (1%)

*From your first incoming class through the graduating class of 2023, how many students did NOT complete the program on schedule, meaning they decelerated or exited the accelerated pathway/program?* Based on the data provided from the institutions, 591 students entered an A3YP; 477 graduated in 3 years; and 114 transitioned out of the A3YP into the traditional 4YP curriculum. Thus, the transition from A3YP to 4YP was 19.2% in total from 2015 through 2023 for the 14 responding schools (95% confidence interval 17–22%) resulting in an overall 3-year graduation rate of 80.8% among A3YP matriculants. As a point of comparison, in 2023, the Association of American Medical Colleges (AAMC) reported that “medical school graduation rates for non-dual degree MD students remained stable from 1998–1999 through 2017–2018” [16]. The 4-year graduation rate ranged from 81.7 to 84.1% [16] per data from the website. Thus, the A3YP graduation rate is comparable to the overall AAMC reported rate for 4-year programs.

The survey also identified reasons for transition from A3YP to traditional programs with the question: *For the students who decelerated or exited the pathway/program, please select the reason why AND number of students per reason. Personal Reasons, Academic Reasons, Specialty Selection, Professionalism, Other.* Of the responses, academic challenges were the most commonly noted reason, accounting for 45 students or 39% of the 114 students who transitioned out of an A3YP. Some common reasons why a student might encounter academic issues that may necessitate transition out of A3YP include failure of US Medical Licensing Examination (USMLE), or a course or clerkship failure. Academic challenges which require remediation can prolong UME training and make it difficult to complete an A3YP logistically; therefore, most students need to transition to 4YP. Transition out of A3YP due to a change in specialty choice was noted for 34 students (30%). Another 27 students (24%) were reported to have transitioned for personal reasons. Professionalism issues accounted for seven students’ transition out of A3YP (6%). It is important to note that some of these reasons for transition out of A3YP might overlap, such as when students face medical illness or personal difficulties that also impact academic performance or exam readiness resulting in an academic issue or resulting in professionalism concerns.

It is of note that our surveyed school’s cohort size varies, ranging from four to 150 total graduates over the duration of their programs’ history. A further limitation is that data is reported as aggregate from all schools that participated to eliminate any potential confidentiality issues. Due to this aggregate reporting the ability to describe whether similar rates occurred in schools with only one career pathway vs several career pathways is not possible and a future study could investigate this question. Reporting on student transitions out of A3YP programs is further complicated by differences in schools’ policies, processes, and internal support. For example, some schools have more stringent promotion guidelines for their A3YP students than for students in 4YP, while others do not. Our discussion focuses on the reasons for transition as well as the processes and guidance required when the transition occurs (Table 2). Schools track the rates and reasons for transition out of A3YP but the sample sizes are small therefore it is difficult to predict the transition rates or trends.

**Table 2:** Guidance for transitioning students to 4-year programs — policies, procedures, and quality improvement

**Policies for A3YP**	
Have a A3YP Policy in place that clearly defines academic, clinical, and professional expectations	• Ensure that the program has defined expectations from the outset. Examples include minimum academic performance on pre-clerkship exams, minimum performance on high stakes summative exams, clinical milestones, participation in parallel curriculum requirements, and professionalism standards • Engage all relevant institutional stakeholders in the development and review of policies, including the school’s curriculum or executive committee, especially when necessary to align procedures between 3-year and 4-year programs • Review program standards on a regular schedule
Ensure clear communication of standards to students	• Clearly outline and communicate program standards and expectations at admission and/or matriculation • Provide written and/or web-based academic policies and procedures that are easily accessible to students, program faculty and staff, and stakeholder committees • At each transitional phase in the curriculum or student progression, make time to review standards to ensure continuous student awareness
Ensure clear communication with faculty involved in A3YP students training	• Conduct at least annually faculty and staff development to review program outcomes and standards for progression. Involve the full complement of advisors, clinical preceptors, learning specialists, and others who facilitate student progression • Provide regular updates to faculty and staff about changes to standards • Empower those involved in student training to provide support and reflections about student performance. Encourage members of the entire team to help with early identification of problems, resource needs, and opportunities for additional support
Establish a A3YP Committee on Transitions	• Establish a Committee on Transitions that can facilitate processes and ensure consistent interpretation of standards; this committee may be school- or program-specific or could include sub-committees if necessitated by the availability of many differing programs • Empower the Committee to assist with independent review of any student being considered for transition from the A3YP program • Develop committee guidelines that address conflict of interests in participant membership and diversity of membership to ensure fairness and unbiased approach to student review. For example, potential sources of bias might arise if GME or program directors are represented or if people in a support position for a student have voting or decision-making in this process
**Procedures to facilitate transition from 3YAP**	
Establish a process for reentering the 4-year MD curriculum	• Identify challenges that may need to be addressed based on the timing of a transition. A student may choose or need to move from a A3YP program at any time in the curriculum; therefore, each student should be provided individualized advice depending on their circumstances • Develop a process to determine how coursework already completed in the A3YP program will be counted toward graduation in the 4-year program • Determine which experiences not included in a A3YP will be required of a student transitioning to a 4-year program. Examples include electives and coursework required of 4-year students
Ensure student support during the transition	• For each individual student, determine what support or resources are needed. Student needs and support may depend on when a student transitions into the 4-year program and the reasons for transitioning • Assist students transitioning out of A3YP due to academic challenges in determining adjusted examination or coursework schedules, academic resources or counseling, or advisors • Connect students with advisors within a new specialty choice
**Quality improvement: Review transitions out of 3AYP**	
Establish a process for quality improvement	• Perform regular reviews of policies and communications that assist in the transition of students into the 4-year MD program to ensure that they are updated and continue to reflect the mission and goals of the program • Identify and address any unanticipated adverse impact on students and adjust standards as needed
Residency involvement	• Keep directed pathway residency programs informed about transition decisions, especially in the lead-up to residency match • Review and follow standards set forth by the Family Educational Rights and Privacy Act (FERPA) [28] regarding when and how reasons for transition can be communicated. The issue of feedforward is key to this discussion — the transition to residency should have some discussion of strengths and opportunities, but in a traditional student this would not come prior to match day

## Balancing Student and Program Support

Transitioning from an A3YP to a traditional 4-year track can be challenging for students, the school, and the directed pathway residency programs. Students may need to take additional coursework or research time to prepare for a possible change in residency specialty. The school may need to schedule additional rotations out of sequence for A3YP students transitioning to a 4YP. These transitions can also cause disruption for the residency programs. For instance, a residency program may have invested in mentoring an accelerated student who now will not be joining their program, possibly leading programs to be reluctant to participate in 3YAP in the future. The majority of these transitions happen before Match, allowing programs to fill the position, but can incur additional time and energy for recruiting and interviewing for the open position.

Despite these challenges, it remains essential that students receive the support and guidance they need to be successful in an accelerated medical pathway program. Structures such as meeting all educational objectives, core competencies, and all program requirements must be in place to ensure competent graduates. Processes should assist students when transitioning out of an accelerated program becomes necessary. Safeguarding student success in medical school while meeting societal contracts to graduate competent physicians is a basic tenant in the UME to GME continuum.

## Academic Progression

When students do not meet academic standards, transitioning from the A3YP into the 4YP may be recommended or required. Consistent among programs is that A3YP students have limited time for remediation of poor academic or clinical performance [17, 18]. They also do not have the availability of additional time to prepare for USMLE Step 1 and 2. It is sometimes difficult to predict at admission which students may or may not thrive in an accelerated pathway, as most of their academic experiences still lie ahead. Thus, it remains the responsibility of each accelerated program to have mechanisms in place to ensure that student progression through the program is closely monitored and achieved to ensure competence at graduation. Early identification and intervention when issues arise is of importance to ensure students stay on track. Some schools proactively enroll all accelerated students into direct tutoring to prevent failures or academic issues before they occur. Other programs use mentoring and coaching to ensure students are identified before they fail or struggle [19].

Accelerated students, A3YP program directors, and residency programs are all stakeholders in the need for clear guardrails and policy and procedures to define satisfactory progression through the curriculum.

Many programs in CAMPP have set standards for accelerated programs that are more stringent than 4YP. Examples include policies stating that one failure in the pre-clerkship stage prompts a formal review or that failure of any clinical subject exams in the core clinical clerkship phase necessitates a review of the student progress or even automatic transition out of an A3YP.

Although these enhanced criteria can put additional stress on a candidate, they also ensure that students have clear expectations for successful program completion and guidelines for transitioning out of an accelerated pathway. Acceleration may not be in the best interests of students who encounter academic difficulty, as they may need more time to master the required clinical competencies and to develop the maturity required for future career success. In a pure sense, competency-based education supports both acceleration and deceleration rather than adherence to predetermined timelines for graduation [20–22].

## Change in Specialty Choice

Accelerated programs vary from those that track students into a single specialty to those that prepare students to enter an array of specialty options. Capacity for directed entry into specific GME programs might also be variable. While some programs allow students to identify a specialty later in their training, most programs either recruit students directly into specialty-specific pathways, offer directed pathways to a limited number of associated specialties, or expect students to make choices early, such as during the first year of medical school. As a result, a change in specialty choice or a decision to delay commitment to specialty choice is one reason that students choose to leave an A3YP. Typically, traditional 4YP medical students choose their specialty at a variety of points in the educational program. Their choices may be based on clinical experiences, mentorship, research, and lifestyle. However, exposure to all the medical specialties may not occur until clinical clerkships, most often in the third year of medical school, when A3YP students are actually preparing for and applying for residency. A3YP students who opt for specialty change will almost always need to do so through a 4-year pathway to pursue clinical or research experiences to ensure fit and success within that specialty and competitiveness for the National Residency Matching Program (NRMP) also known as Match.

It should be noted that while 30% of the 114 students in our analysis who transitioned out of A3YP did so because of a change in specialty choice, this represents only 6% of the 591 students who began A3YP training. In contrast, AAMC data indicate that approximately 74% of medical students change their specialty choice from the time of matriculation to the time of graduation [23]. This consistency in A3YP students’ dedication to a specialty choice is likely a reflection of early specialty exposure and mentorship from program faculty and residents, as well as to the commitment that those programs and faculty show to the A3YP students, which are not generally available to traditional pathway students.

## Personal and Medical Issues

When faced with significant personal or medical issues, many students have difficulty focusing on the rigorous requirements of medical education, particularly if illness results in prolonged absence. Greenburg et al. noted that 22% of medical students reported experiencing a major life crisis during medical school in our sample 4% of students transitioned out of the A3YP for personal reasons [24]. While the accelerated program may be compressed, Leong et al. found rates of mean scores on the exhaustion subscale on the AAMC Graduation Questionnaire were similar between A3YP and 4YP students [10].

Examples of personal reasons for transitioning out of the A3YP programs include childbirth-related leave, personal illness, and other family responsibilities. Personal relationships may factor into the decision to transition out of the A3YP program including accommodating a life partner’s career or a desire to train in a different geographic location.

The shortened timeline of an accelerated program leaves little to no room for coping with medical or personal issues and makes them more difficult to navigate, as the pathways by necessity eliminate some vacation and most elective time. Since most programs have a directed pathway into residency with an expected start of July 1, residency programs can rarely accommodate a delayed entry. Therefore, discussion of these program limitations must occur frequently and often for students in an accelerated program so that guidelines are well known. All programs should work to identify solutions to keep a student in the accelerated program, if possible, but always with the best interest of the student as the foremost goal.

## Professionalism Issues

Professionalism, as defined by the American Board of Medical Specialties is “a commitment to carrying out professional responsibilities, adherence to ethical principles and sensitivity to diverse populations” [25]. Medical education encourages the development of professional identity formation (PIF) and exposes students to the values, which include competence, compassion, caring, honesty, and integrity, behaviors, and relationships, which underpin the trust the public has in doctors [26, 27]. Seven students transitioned from A3YP due to professionalism issues which is 6% of the A3YP students who transitioned to 4YP. However, this is only 1% of the entire A3YP cohort of 591. Although this is a very small percentage, it is no less important to recognize that PIF must be addressed and developed in 3 years rather than 4. The UME programs must ensure that the accelerated student has the professional maturity to accept the responsibilities of residency and the tenets of patient care. Professionalism lapses must be carefully monitored for all medical students including those in accelerated pathways. With the abbreviated time frame in accelerated pathways, remediation of professionalism issues may be difficult to accomplish. Policies should be in place to ensure that A3YP program directors are informed of professionalism concerns and are in a position to intervene and remediate early to avoid larger concerns. Programs should decide in advance how to address issues that may arise and have written policies in place regarding the level of concern that would necessitate transition from an A3YP. Since professionalism issues are less quantitative than other metrics, it is helpful to have committee involvement to confer on decisions surrounding professional conduct, remediation, or transition out of A3YP.

## Summary

Transitions out of A3YP programs will occur so it is therefore important to have standard policies and procedures in place. Policies include defined program criteria and circumstances which would necessitate a transition to the 4YP. Standard policies to guide the transition and a mechanism for review of procedures after a transition has occurred are important elements of an A3YP. A successful A3YP program will have standard policies that are clearly communicated to students and all faculty involved in training. Students must be aware of any differences in defining student progress compared to their cohorts in 4YP. Programs should have tutoring, mentoring, and/or coaching in place to ensure success.

When students transition out of an A3YP, it is important to follow standard processes so that students have clear expectations, unbiased support, guidance, and a student-centered approach to the altered curriculum. It is also important to have a process of hand-off in place that involves student affairs personnel so that students who have been in a small cohort and are transitioning into the 4YP continue to meet graduation requirements and are supported by the 4-year medical school faculty in this transition.

All programs should have policies in place that include continuous quality improvement and review reasons for transition at regular intervals. The authors pulled from the collective experience the key factors that were felt were most important for student success, transition, and review process and these are summarized in Table 2.

In conclusion, the on-time graduation rate for accelerated students is 81% in this analysis, demonstrating that 19% of the cohort may choose or need to leave an A3YP. The most common reasons for exiting the A3YPs are academic issues, change in specialty choice, and personal reasons. Future studies can include investigation into student and programmatic factors that lead to success and retention of students.

## Data Availability

Data available on request.

## Appendix

**Table 3:** Schools included

1. Cooper Medical School of Rowan University
2. Medical University of South Carolina
3. Mercer University School of Medicine
4. NYU Grossman SOM
5. Penn State University College of Medicine
6. Renaissance School of Medicine at Stony Brook University
7. Rutgers New Jersey Medical School
8. Texas Tech University Health Sciences Center School of Medicine
9. The Ohio State University College of Medicine
10. University of California Davis School of Medicine
11. University of Louisville Trover Campus
12. University of North Carolina
13. Virginia Commonwealth University
14. West Virginia University School of Medicine
